# Diversity of epiphytic bacterial communities on male and female *Sargassum thunbergii*

**DOI:** 10.1186/s13568-022-01439-1

**Published:** 2022-07-16

**Authors:** Jing Wang, Zhibo Yang, Gaoge Wang, Shuai Shang, Xuexi Tang, Hui Xiao

**Affiliations:** 1 College of Marine Life Sciences, Ocean University of China, Qingdao, 266003 China; 2 College of Biological and Environmental Engineering, Binzhou University, Binzhou, 256600 China; 3 Laboratory for Marine Ecology and Environmental Science, Qingdao National Laboratory for Marine Science and Technology, Qingdao, 266000 China

**Keywords:** Diversity, Epiphytic bacteria, Male and female, *Sargassum thunbergii*, 16S rDNA high-throughput sequencing

## Abstract

The epiphytic bacteria are the most abundant microorganisms on marine macroalga. However, there are few studies on the distribution of these epiphytic bacteria on male and female *Sargassum thunbergii*. In this study, the composition and diversity of epiphytic bacterial communities on male and female *S. thunbergii* were investigated by using the traditional culture-based method and 16S rDNA high-throughput sequencing. The results showed that the dominant bacterial phyla and genera were the same on both male and female *S. thunbergii*. However, there were significant differences in the relative abundance of epiphytic bacteria at the genus level. Furthermore, male and female *S. thunbergii* had their own indicative species and specific bacteria. In addition, the predicted functions of the epiphytic bacteria mainly differed in transport and metabolism, environmental adaptation and spore development. This study enriches the baseline knowledge of epiphytic bacteria related to dioecious algae and paves the way for further studies of the relationships between epiphytic microbial communities and the sex of algae.

**Supplementary Information:**

The online version contains supplementary material available at 10.1186/s13568-022-01439-1.

## Introduction

A large number of bacteria adhered to the surface of marine macroalga have multifaceted and complicated interactions with their host macroalgae (Selvarajan et al. 2019). The interactions are not just limited to the nutritional supply of macroalgae to bacteria (Croft et al. 2005). Bedsides, macroalga can suppress the growth of microbes by releasing antibacterial substances (Campbell et al. 2015) and this would affect the epiphytic bacterial community. Meanwhile, epiphytic bacteria can promote the growth and morphological development of macroalgae by producing specific extracellular products (Florez et al. 2017). However, thick biofilms formed by epiphytic bacteria may lead to a reduction in algae productivity (Mathai et al. 2018), and some epiphytic bacteria are potential pathogens that can destroy macroalgal cells or even cause algal death (Egan et al. 2014). The result of mentioned interactions would give macroalgae and bacteria this capability to select the appropriate ones for a possible symbiosis. This would result in a specific epiphytic bacterial community on macroalgae.

There are many reports on the community structure of epiphytic bacteria on macroalgae, most of which pay attention to their community changes in different phyla, families, and genera of host-macroalgae (Florez et al. 2017), as well as different growth stages, different parts or different health states of the same host, etc. (Serebryakova et al. 2018). Those changes can be explained by the specific polysaccharides of the cell wall (such as agar, carrageenan, and alginate) in macroalga (Popper et al. 2011) and the ability of epiphytic microorganisms to produce specific degradation enzymes such as dehalogenases, antimicrobials, and alga-specific polysaccharidases (e.g., agarases, carrageenases, and alginate lyases) (Martin et al. 2014). In addition, the secondary metabolites produced and secreted by different macroalgae can selectively attract or repel specific bacteria (Collén and Davison 2001). For example, some novel antibacterial lactones, such as macrolactines G-M, may prevent the colonization of some bacterial communities (Florez et al. 2017) while other such molecules may favor the settlement of different microorganisms (Florez et al. 2017). Therefore, the characteristics of the macroalga may be key to determining the epiphytic bacterial community structure.

Among dioecious algae, male and female individuals show sexual differences in morphology, cell structure, physiology, and biochemistry (such as stomatal conductance, net CO_2_ assimilation rate, abscisic acid levels in leaves, and freezing tolerance) (Liao et al. 2020; Tang 2020). The differences of epiphytic bacteria on dioecious higher plants are better known; for example, the epiphytic bacterial communities in *Populus cathayana* rhizospheres differ between the sexes (Liu et al. 2020). Additionally, some bacteria were differentially enriched on the male and female *Porphyra haitanensis* (Yang et al. 2022). Epiphytic and specific bacterial communities associated with the sex of the macroalgae need further research. The analysis of the differences between epiphytic bacterial communities of dioecious algae is the basis of the above research.

Macroalgae is an important component of marine ecosystem. *Sargassum thunbergii*, a very important genus of brown algae, is critical to maintain coastal ecosystem and provide residence/refugees for marine animals (Amaral-Zettler et al. 2016). *S. thunbergii* is a common intertidal species in northern China (Xu et al. 2021). As an important economic alga, *S. thunbergii* is an industrial raw material and high-quality bait for cultivated abalones and sea cucumbers. At the same time, *S. thunbergii* plays an important role in nutrient regulation and habitat restoration (Wu et al. 2010). The ecological research on *S. thunbergii* is of great significance to the protection and restoration of *S. thunbergii* resources and the development of *S. thunbergii* cultivating industries.

As dioecious algae, male and female *S. thunbergii* can essentially be distinguished from morphological structures. Receptacles on males are slender and smooth, while receptacles on females are short and coarse (Wang and Liu 2007). Differences in morphological structure on male and female *S. thunbergii* may result in physiological and biochemical differences. Thus this may result in presence of different epiphytic bacteria on male and female *S. thunbergii*. In this study, the composition and diversity of epiphytic bacterial communities of both male and female *S. thunbergii* were investigated by both 16S rDNA high-throughput sequencing and culture-based methods*.* The results provide a basis and enlightenment for further understanding the differences in epiphytic bacteria between male and female *S. thunbergii*, as well as the interaction between marine macroalgae and their epiphytic bacteria.

## Materials and methods

### Sampling site and sampling male and female *S. thunbergii*

The sampling site was located in the rocky intertidal zone (36° N, 120° E) in Qingdao (Shandong, China) in July 2019. Samples (with a similar habitat, that displayed excellent growth, that were approximately 10 cm in height and about 8 g in weight, that were intact, and that had no spots caused by disease or insect pests) were collected from the same site and placed in sterilized sealed bags with sterile gloves. Male and female *S. thunbergii* were identified by morphological observation under a microscope (Nikon H600L, Tokyo, Japan).

### Samples of epiphytic bacteria on male and female *S. thunbergii*

Epiphytic bacteria were sampled as previously described (Kembel and Mueller 2014; Mathai et al. 2018) with slight modifications. Briefly, 25 g of male and female *S. thunbergii* (three groups per sex), of similar shape and size, were weighed and placed in sterile Erlenmeyer flasks. Then, 70 mL of sterile phosphate-buffered saline (PBS) buffer (1 mmol/L) was added to each flask and the flask was sealed with a sterile membrane. Next, the flask was shaken (200 R·min^−1^) for 30 min at room temperature to obtain a suspension of epiphytic bacteria. The bacterial suspension was filtered through sterile gauze (10 cm × 10 cm); then, the epiphytic bacteria were collected by vacuum filtration through the 0.22-μm microfiltration membrane. Further, a positive and a negative control per experimental setup were included.

### Isolation, purification, and identification of culturable heterotrophic bacteria

Culturable heterotrophic bacteria were isolated on Zobell 2216E solid agar plates by the culture-based method described previously (Vendan et al. 2010). DNA was extracted using an Ezup Column Bacterial Genomic DNA Purification Kit (Sangon Biotech, Shanghai, China) following the manufacturer’s instructions. Then, 16S rDNA sequences were amplified using universal primers 27F and 1492R and sequenced at Sangon Biotech (Shanghai, China). PCR products were sequenced and analysed with the NCBI BLAST program to obtain the most similar standard strain of the tested strain.

### 16S rDNA high-throughput sequencing

Total DNA of epiphytic bacteria was extracted with the E.Z.N.A. ® Stool DNA Kit (Omega Biotek, USA). Amplicon synthesis, library construction, and Illumina NovaSeq PE250 platform sequencing were conducted at LC-BIO Technologies Co., Ltd. (Hangzhou, China). Amplification of the V4 region of bacterial 16S rDNA gene using universal primers 515F and 806R Paired-end 16S sequences were assembled using FLASH (v.1.2.7). Then, the sequences were quality-trimmed and length-filtered in fqtrim (v0.94). Chimeric sequences were filtered using VSEARCH software (v2.3.4). DADA2 was used for quality filtering, denoising, paired-end merging and ASV assignment, and each ASV was identified to the appropriate taxon using the QIIME 2 plugin. In this process, the mitochondrial sequence, unclassified kingdom, *S. thunbergii* sequence and chloroplast sequences were filtered out, and chimaeras were removed.

### Data analysis

Data analysis was performed based on methods described in a previous study (Mathai et al. 2018). The α-diversity indices (Shannon index, Simpson index, Fisher alpha, and Chao1) were tested using analysis of variance (ANOVA) and β-Diversity was calculated using unweighted UniFrac distances via principal coordinate analyses (PCoA: ANOSIM test). The analysis was performed separately for the 3 groups (All; Abundant taxa; rare taxa). The definition of abundant and rare taxa depends on the cutoff level of relative abundance, setting 0.01% as rare ASVs and 1% as abundant ASVs (Xue et al. 2018). All indicators were calculated with QIIME2, and analyses were performed in R software (v3.6.1). DESeq2 were used to analyse differential ASV. Blast was used for sequence alignment, and the feature sequences were annotated with SILVA (Release 132) and NT-16S databases for each representative sequence. Taxonomic summaries were performed by calculating the relative abundance across samples and normalizing to 100% (Li et al. 2021). LEfSe combines Kruskal–Wallis test or pairwise Wilcoxon rank-sum test with linear discriminant analysis (LDA) were applied to obtain the final differential species (i.e., biomarker) (*P* < 0.05, LDA SCORE > 3). The PICRUSt2 package with the KEGG Orthology (KO) databases was used to predict the metagenome functional context, and then t-test was used to test the significance level of comparisons across samples. Afterward, they were drawn in R software using “pheatmap” package. Univariate and multivariate statistical analyses were performed using SPSS 18.0 software.

## Results

### Epiphytic bacterial communities on male and female *S. thunbergii* as determined using the culture-based method

**Fig. 1:**
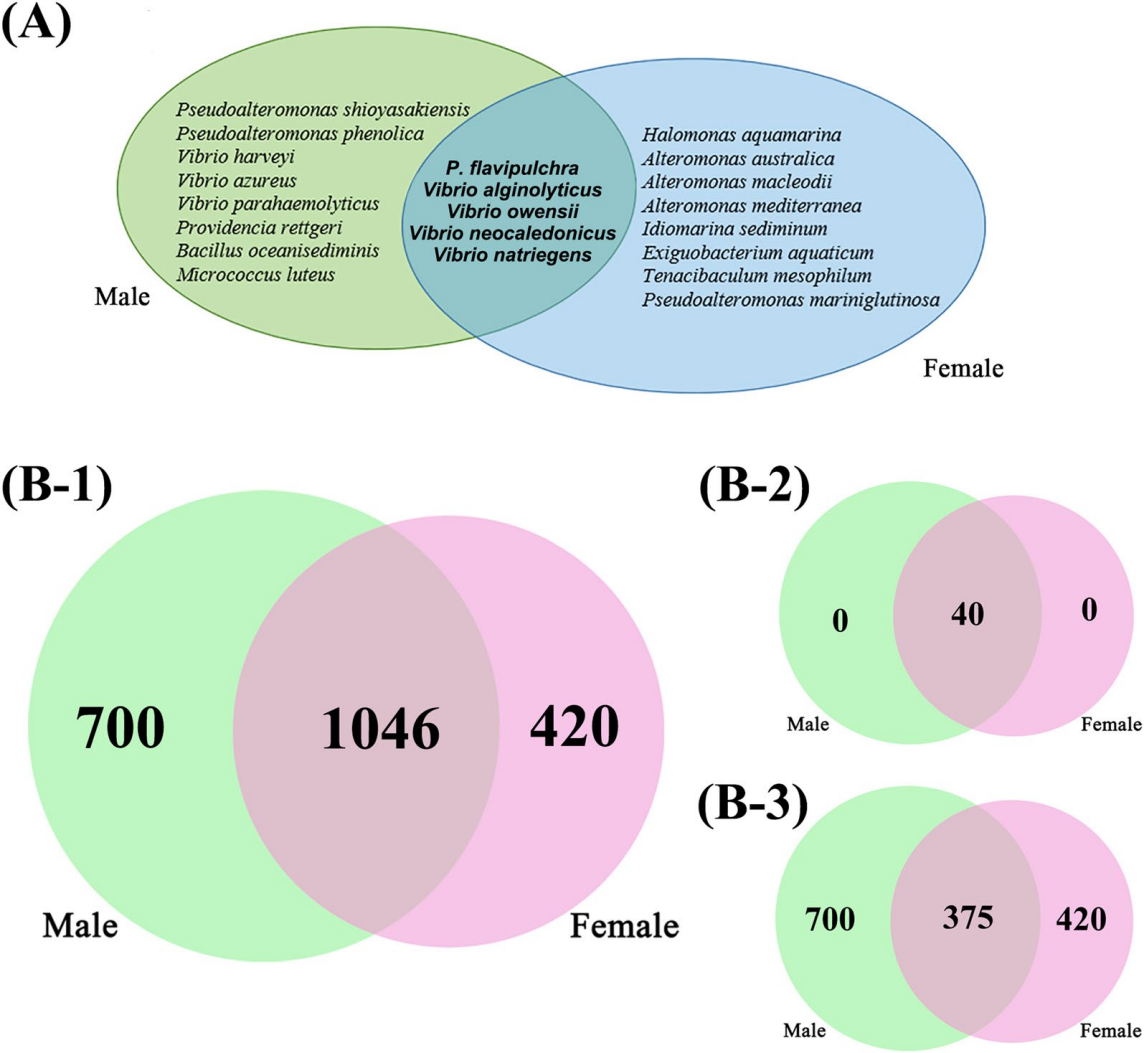
Venn diagram of **A** culturable heterotrophic epiphytic bacteria on male and female *S. thunbergii* at the species level. **B** Numbers indicate numbers of **B-1** all the ASVs of epiphytic bacteria on male and female *S. thunbergii*, **B-2** abundant epiphytic bacteria on male and female *S. thunbergii*, **B-3** rare epiphytic bacteria on male and female *S. thunbergii*

A total of 63 strains of culturable aerobic heterotrophic bacteria were isolated from male and female *S. thunbergii* and were identified as belonging to 4 phyla, 11 genera, 22 species and 1 potentially novel species (92.84% 16S rDNA similarity). The identification results were shown in Additional file 1: Table S1. Five species were found in male and female *S. thunbergii*, including *Pseudoalteromonas flavipulchra, Vibrio alginolyticus*, *Vibrio owensii*, *Vibrio neocaledonicus* and *Vibrio natriegens* (Fig. 1A).

### Sequencing summary by 16S rDNA high-throughput sequencing

2166 different ASVs were clustered. Good's coverage for all samples was higher than 0.99, and the rarefaction curves of all samples tended to be saturated with increased sequencing amounts, indicating that the sequencing depth could cover most species of the samples and could be used for further data analysis (Additional file 1: Fig. S1). Of these ASVs, 1046 were shared by both male and female *S. thunbergii*. Overall, 700 and 420 ASVs were specific to male and female *S. thunbergii*, respectively (Fig. 1B-1).

### *α-diversity* and *β-diversity*

Analysis of the α-diversity of all the epiphytic bacteria on male and female *S. thunbergii* revealed that there was no significant difference (*P* > 0.05) between female and male *S. thunbergii* (Table 1)*.* However, *α-*diversity of abundant taxa (Shannon and Simpon) and rare taxa (Fisher) showed significant difference (*P *< 0.05) in microbiome diversity between the males and the females (Table 1).

**Table 1:** Alpha-diversity indices of the epiphytic bacteria on both male and female *S. thunbergii*

	All			Abundant			Rare		
	Male	Female	*P*	Male	Female	*P*	Male	Female	*P*
Chao1	1182	979	0.078				429	274	0.052
Shannon	8.510	8.360	0.359	3.380	3.460	0.009^**^	5.669	5.267	0.065
Simpson	0.991	0.993	0.291	0.951	0.960	0.013^*^	0.994	0.992	0.192
Fisher	183.885	149.064	0.067	4.743	4.683	0.411	110.966	77.048	0.045^*^

*P < 0.05 reflects significant difference, and **P < 0.01 reflects extremely significant difference

Abundant ASVs were persistent across all samples, and the values and *P* values of Chao1 indices cannot be calculated

There was no significant difference in β-diversity index (*P* > 0.05) between male and female *S. thunbergii* (Additional file 1: Fig. S2), but the bacterial communities on the male *S. thunbergii* clustered separately from that on female (Additional file 1: Fig. S2).

#### Analysis of abundances of differential ASVs

**Fig. 2:**
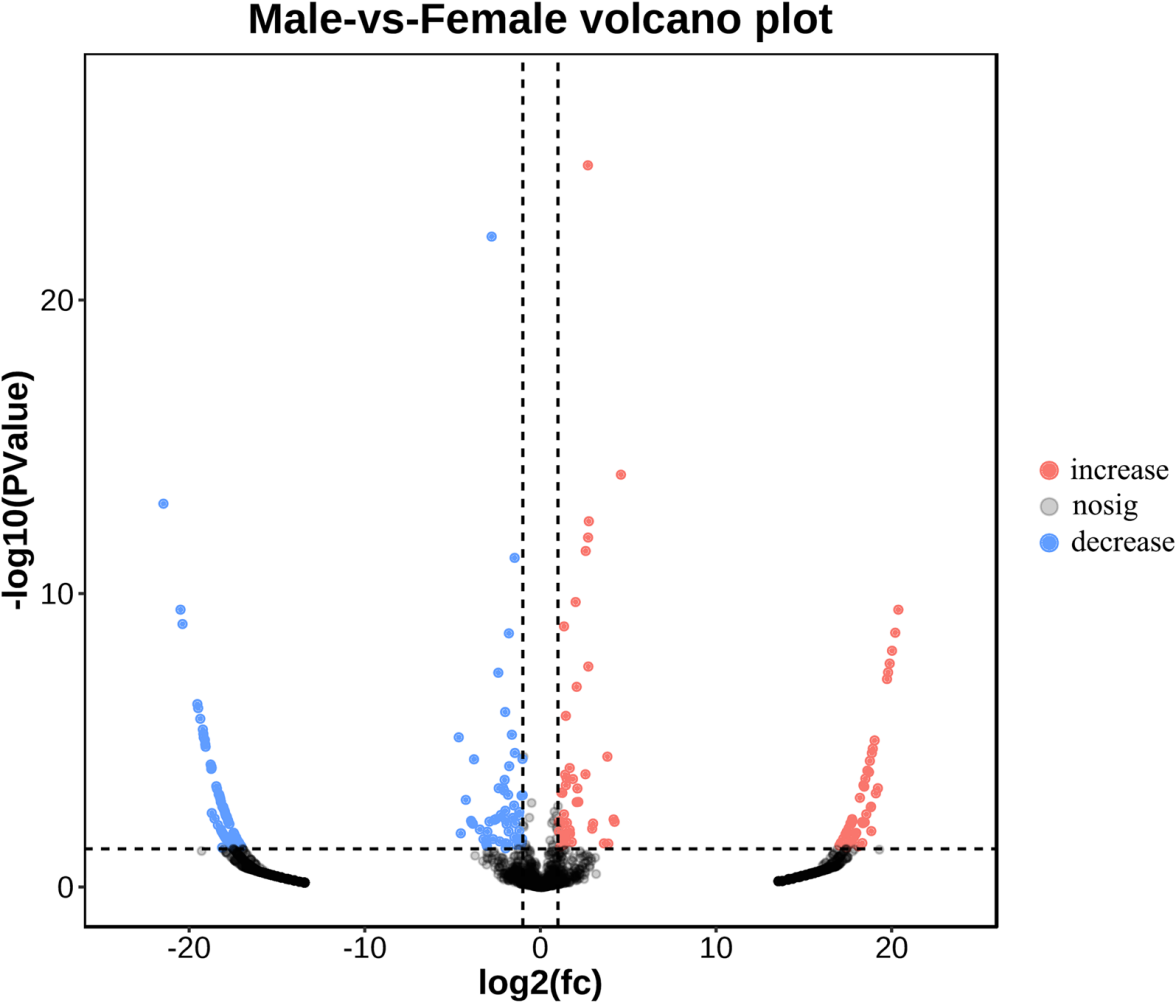
Volcano plot displaying the increased and decreased relative abundance of ASVs associated with male and female *S. thunbergii*. The *P* values were calculated based on DEseq using a negative binomial distribution. The y-axis represents the mean relative abundance value of log10 (*P* value, t test), and the x-axis displays the log2-fold value. The red dots represent the ASVs with increased relative abundance (n = 92, *P* < 0.05) of female *S. thunbergii*, and the blue dots represent the ASVs with decreased relative abundance compared to those of male *S. thunbergii*. (n = 139, *P* < 0.05). Log2(fc) cut-off: 2

DESeq2 analysis showed that there was a significant difference (*P* < 0.05, using a negative binomial distribution) in the relative abundances of epiphytic bacteria between male and female *S. thunbergii* (Fig. 2). The relative abundance of 1936 ASVs was not significantly different between the male and female groups (*P* > 0.05). Compared with the relative abundances of the ASVs on male *S. thunbergii*, the relative abundances of 92 and 139 ASVs on female algae significantly increased (*P* < 0.05) and decreased, respectively (*P* < 0.05).

#### Community structure of the epiphytic bacterial community

**Fig. 3:**
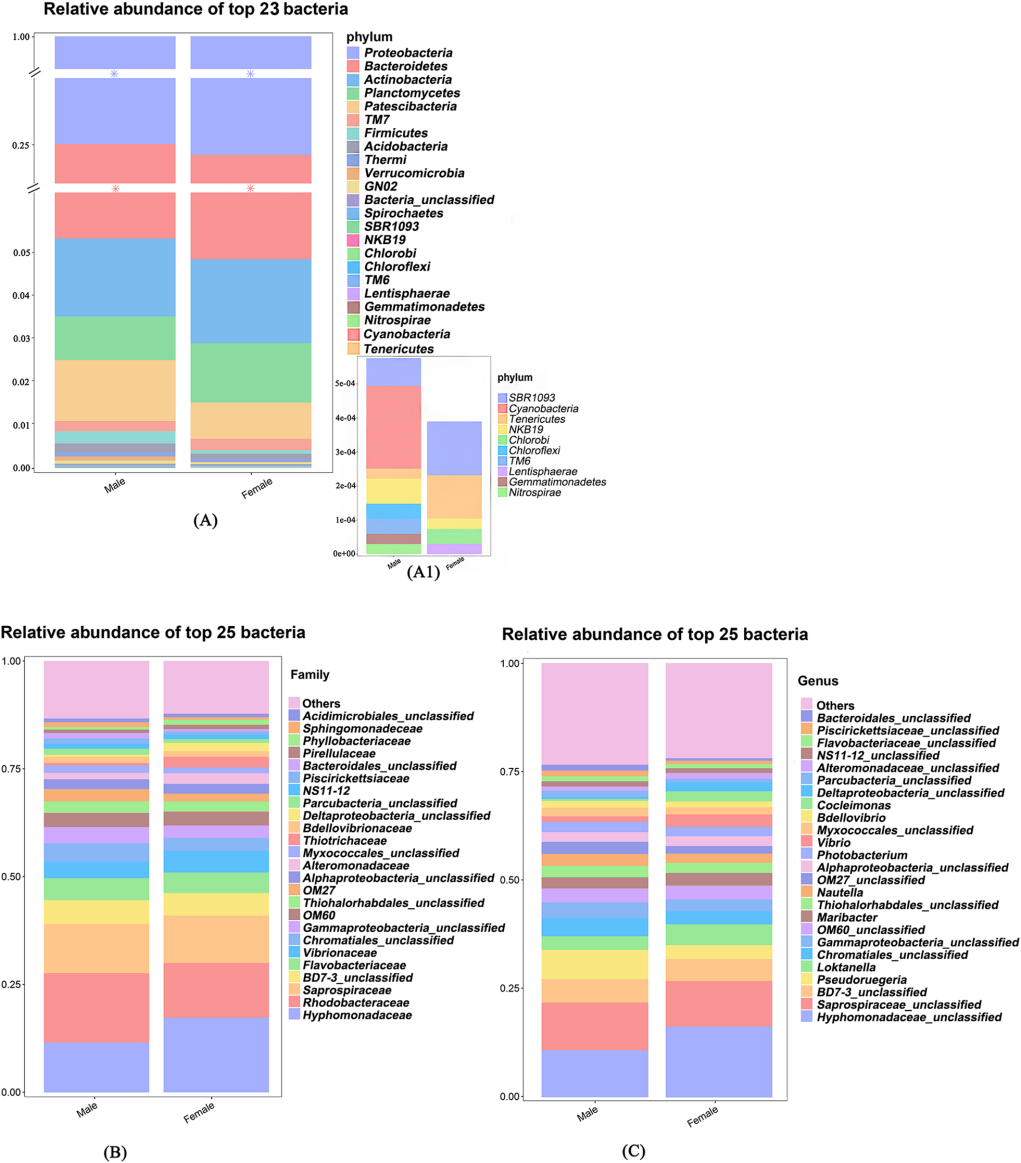
Composition of epiphytic bacteria of male and female *S. thunbergii*. **A** Phylum level; **B** family level; **C** genus level

Based on the comparation by relative abundance (Table 2, Fig. 3), most of the epiphytic bacteria were common to male and female *S. thunbergii*. There were 21 phyla, 41 classes, and 184 genera on male *S. thunbergii*, and 18 phyla, 36 classes, and 169 genera of bacteria on female *S. thunbergii* (Table 2). However, the relative abundances of epiphytic bacteria on male and female *S. thunbergii* differed (Additional file 1: Table S2). At the phylum level, *Proteobacteria* was common to both male and female *S. thunbergii* (accounting for 74.75% on male and 77.31% on female *S. thunbergii*, respectively). The overall dominant genus (*Pseudoruegeria*) was shared between sexes and constituted 6.88% and 3.27% of the reads (Table 3).

**Table 2:** Composition of the epiphytic bacterial ASVs of male and female *S. thunbergii*

Sample	Phylum	Class	Order	Family	Genus
Male	21	41	73	124	184
Female	18	36	67	111	169
Features sequences of both sexes	16	31	61	103	145
Only found on males	5	10	12	21	39
Only found on females	2	5	6	8	24
Total	23	46	79	132	208

**Table 3:** Relative abundance of dominant epiphytic bacteria and male- and female-specific bacteria at the genus level

Genus	Male (%)	Female (%)
*Hyphomonadaceae_unclassified*	11.08 ± 2.00	16.84 ± 1.80
*Saprospiraceae_unclassified*	11.18 ± 1.50	10.69 ± 1.20
*BD7-3_unclassified*	5.50 ± 0.30	5.16 ± 0.60
*Pseudoruegeria*	6.88 ± 0.40	3.28 ± 0.20
*Loktanella*	3.18 ± 0.10	4.90 ± 0.15
*Polaribacter*	0.2298 ± 0.01	0
*Croceitalea*	0.0715 ± 0.01	0
*Coxiella*	0.0581 ± 0.01	0
*Reichenbachiella*	0	0.0556 ± 0.01
*Labrenzia*	0	0.0425 ± 0.01
*Spongiibacter*	0	0.0387 ± 0.01

Performed by calculating the relative abundance across samples and normalizing to 100%. Values are expressed as the mean ± SD (standard deviation)

Male- and female-specific bacteria were identified on *S. thunbergii* and could be found in all of the replicates of this condition. However, the proportions were very low (0.94% for males and 0.41% for females) (Additional file 1: Table S3). Male *S. thunbergii* had more specific epiphytic bacterial genera, with a higher proportion than did female *S. thunbergii* (Table 2). Table 3 lists the top bacteria shared by both sexes as well as male- and female-specific bacteria. *Polaribacter* (0.2298%) and *Reichenbachiella* (0.0556%) had the highest relative abundances of male-specific and female-specific bacteria, respectively.

#### Analysis of indicative species

**Fig. 4:**
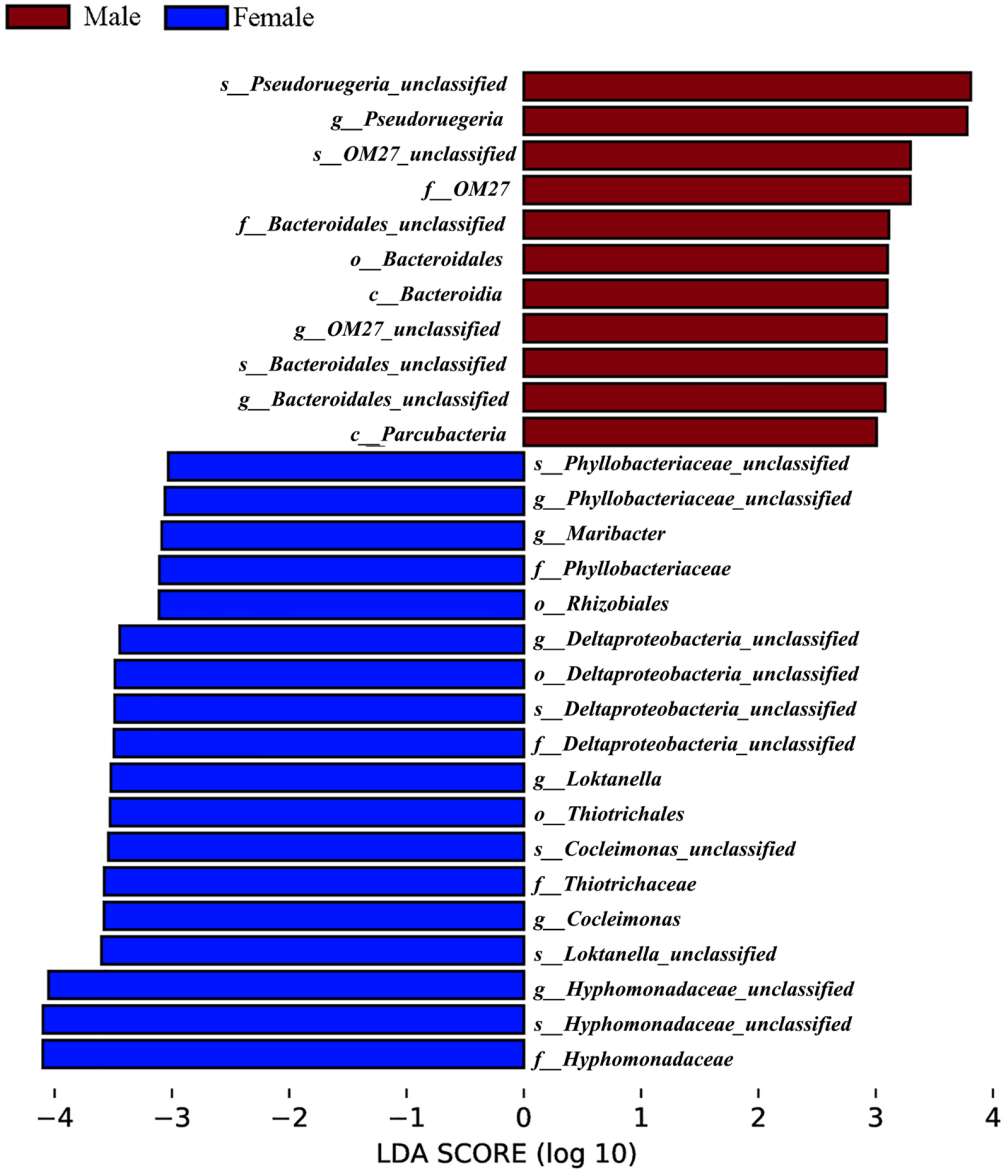
Indicative taxa for male and female *S. thunbergii*. A forest plot showing taxa that were significantly differentially abundant between the male (red) and female (blue) groups as determined using the Kruskal–Wallis test. LDA score (effect size) indicating significant differences in bacterial taxa (P‐value: Wilcoxon rank‐sum test, LDA score > 3.0)

The results showed that there were 11 and 18 indicative epiphytic bacterial taxa on male and female *S. thunbergii*, respectively (Fig. 4). Among them, the genus *Pseudoruegeria*, family *OM27*, and order *Bacteroidales* were significantly enriched in the male group, while the family *Hyphomonadaceae*, the genera *Loktanella* and *Cocleimonas* were enriched in the female group. In addition, bacteria from the genus *Pseudoruegeria* and the family *Hyphomonadaceae* showed enrichment, with the highest LDA scores for male and female *S. thunbergii*, respectively.

#### Predicted functions of epiphytic bacteria

**Fig. 5:**
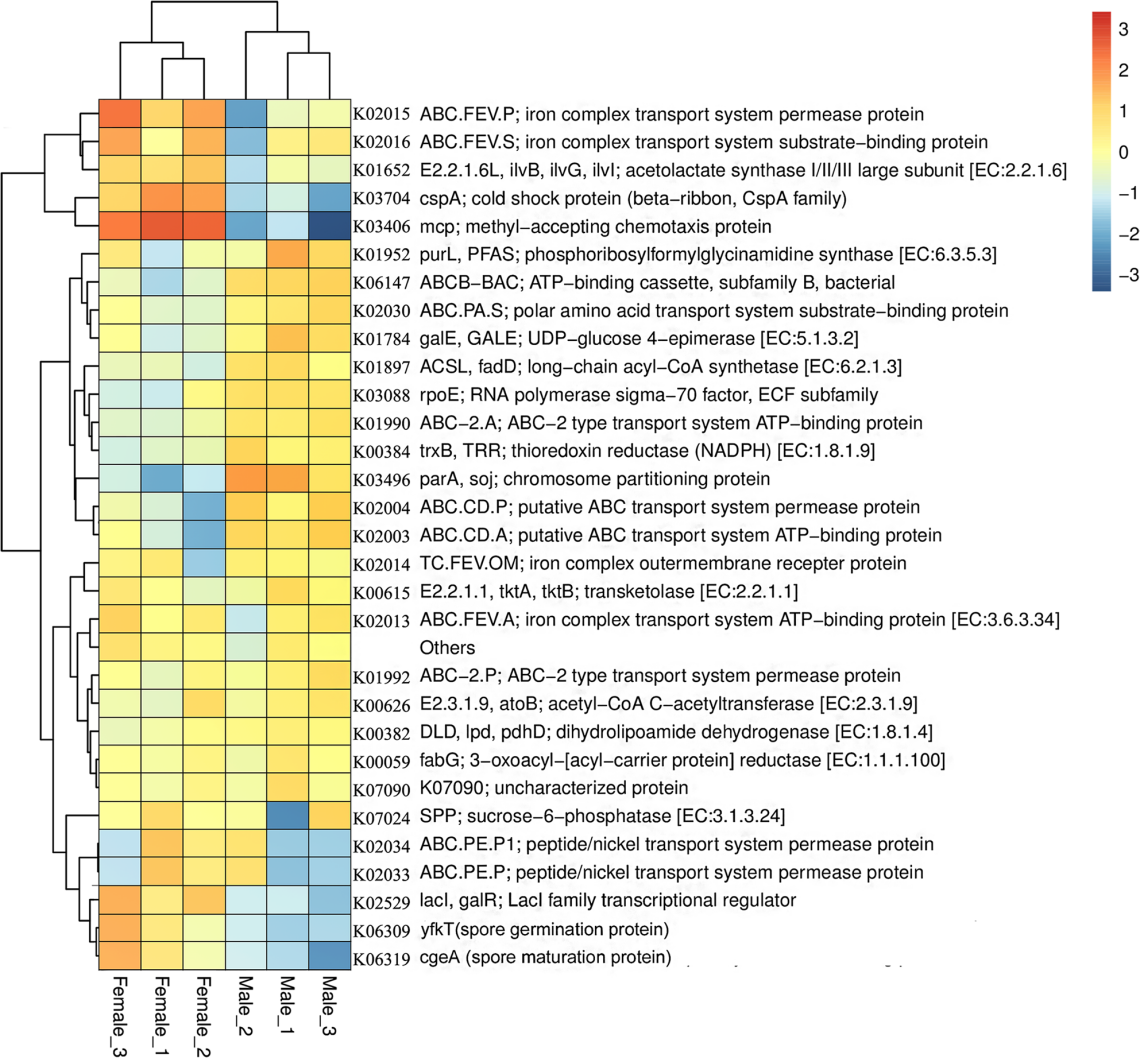
Predicted functions of epiphytic bacteria on male and female *S. thunbergii*. Heatmap of predicted microbial functions by PICRUST2

The predicted functions of bacteria on male and female *S. thunbergii* were mostly the same, and mainly involved nutrient synthesis and operation of physiological functions. However, many genes were different between samples from male and female *S. thunbergii* (Fig. 5). First, genes that perform the same function have different types and abundances. The functions of some epiphytic bacteria on male *S. thunbergii* offered advantages of transport and metabolism because there were six kinds of predicted transport genes from male *S. thunbergii*, but only four kinds from female *S. thunbergii*. Regarding the function of nutrient synthesis and metabolism, there were three functional genes from male *S. thunbergii,* but only one from female *S. thunbergii*.

Additionally, the epiphytic bacteria on male and female *S. thunbergii* also have some sex-specific functions for environmental adaptation. The samples on male *S. thunbergii* have the predicted gene which plays an important role in maintaining cellular redox balance and environmental stress, while the samples on female *S. thunbergii* have the predicted genes related to adaptation to low-temperature environments and to the biodegradation of pollutants, respectively.

Notably, the abundances of proteins related to spore development varied greatly between samples on male and female *S. thunbergii*. The predicted genes, *Yfkt* and *cgeA*, which promote spore development and maturation, respectively, were only abundant in samples from female *S. thunbergii*. In contrast, *parA, soj*, which is related to inhibiting spore formation, was enriched in samples from male *S. thunbergii*.

## Discussion

There was no significant difference in the community composition, and dominant taxa of epiphytic bacteria between male and female *S. thunbergii*. The results indicated that sex did not greatly affect epiphytic bacterial communities on *S. thunbergii*. The same result was observed in marine macroalga *Agarophyton vermiculophyllum* (Bonthond et al. 2020) and *P. haitanensis* (Yang et al. 2022) that showed the sex had only a negligible, statistically insignificant impact on the bacterial composition. However, we found that the relative abundance of some genera, indicative species, specific bacteria and predicted functions of epiphytic bacteria were different between male and female *S. thunbergii*. This result was consistent with a previous study that reported a significant difference in the relative abundance of phyllosphere bacterial and fungal communities at the genus level between male and female *P. cathayana* (Liu et al. 2020). Therefore, we speculate that sex has a certain impact on the epiphytic bacterial community of *S. thunbergii*.

The results obtained with both methods were mutually consistent, and male and female *S. thunbergii* shared most of the epiphytic bacteria. On the one hand, the shared genera, including *Pseudoalteromonas* and *Vibrio,* which were found by both methods, as well as the isolated *Flavobacterium*, *Halomonas* and *Bacillus,* along with *Loktanella* and *Maribacter,* which were identified by 16S rDNA high-throughput sequencing, have been reported to be related to growth and development (Goecke et al. 2010). Among them, *Pseudoalteromonas* strains showed a range of effects, including settlement-inhibiting, paralyzing, and lysing activities, while some strains belonging to the genus *Vibrio* could degrade algal compounds and showed pathogenicity (Florez et al. 2017). In addition, enzymatic activities have been detected in *Flavobacterium* spp., *Halomonas* spp., and *Bacillus* spp. that are relevant to the degradation of macroalgal cell walls. On the other hand, the community structure of marine algal epiphytic bacteria is closely related to the host species (Serebryakova et al. 2018). In this study, *Proteobacteria* was the most dominant bacterium, which is consistent with the results of previous studies showing that *Proteobacteria* was the most dominant bacterium in all seaweeds (Florez et al. 2017). The dominant phyla *Acidobacteria* and *Bacteroidetes* were also found to be dominant taxa for *Heterokontophyta* and *Rhodophyta* (Florez et al. 2017). *Gemmatimonadetes* was specific in the *Heterokontophyta*. All of these results are consistent with previous reports (Florez et al. 2017). However, it is important to mention that *Patescibacteria* has not been reported in *Heterokontophyta* before (Serebryakova et al. 2018), and whether this phylum is specific to *S. thunbergii* will require more experimentation for support.

The highest proportions of male- and female-specific bacteria were *Polaribacter* and *Reichenbachiella,* respectively (Table 2), but it is interesting that both of them are reported to degrade polysaccharides (Wietz et al. 2015; Xing et al. 2015). Additionally, most of the specific bacteria have their own functions. On male *S. thunbergii*, it has been reported that some bacteria of the dominant male-specific genus *Polaribacter* are capable of degrading agar in macroalgae, and *Polaribacter* is dominant on *Gracilaria lemaneiformis* (Hu et al. 2017). *Polaribacter* sp. can also trigger complete morphogenesis of *Ulva* algae alone, which is a newly discovered phenomenon involved in bacteria-induced algal development (Grueneberg et al. 2016). The dominant specific bacterial genus *Labrenzia* on female *S. thunbergii* exhibits many functions, such as participating in the nitrogen cycle in the atmosphere and promoting algal biomass accumulation, growth rate, organic matter degradation, and resistance to heavy metals, antibiotics, and other toxic compounds (Amiri Moghaddam et al. 2018). There are few reports on whether the specific bacteria on dioecious macroalgae are determined by sex, a possibility that needs to be further studied.

The indicative species with the highest LDA values for male and female *S. thunbergii* were the genus *Pseudoruegeria* (male *S. thunbergii*) and the family *Hyphomonadaceae* (female *S. thunbergii*), respectively. *Pseudoruegeria*, which is known to exist in algal surroundings (especially dinoflagellates) or on dead algae, can use dimethylsulfoniopropionate (DMSP) released by algae as a source of sulfur and carbon for growth and is capable of degrading and metabolizing sulfides (Saha et al. 2012). *Hyphomonadaceae* can remove nitrogen, secrete tryptophan and protein, and contribute to microbial aggregation (Weigel and Pfister 2019). These indicative bacteria have different functions, but whether these functions are consistent with the growth and metabolic activities of male and female *S. thunbergii* needs more experiments for verification.

The abundance of predicted functional genes mainly differed in functions related to transport and metabolism, environmental adaptation and spore development between male and female *S. thunbergii*. First, the predicted functional genes regarding nutrient transport, synthesis and metabolism of the epiphytic bacteria on male *S. thunbergii* were more abundant than that on female *S. thunbergii.* In plants, males can make better use of nutrients and grow better than females (Barrett and Hough 2013). Whether this difference also exists between male and female *S. thunbergii*? The increased material transport and metabolism of male *S. thunbergii* can lead to the expression of related functions of epiphytic bacteria using host metabolites. However, due to the lack of data on male and female *S. thunbergii*, this speculation needs to be further verified. In addition, the expression levels of the iron complex transport system and the peptide/nickel transport system were abundant in the epiphytic bacteria of female *S. thunbergii.* These functions have been reported to increase the mineral contents of roots and aboveground tissues and increase tolerance to metal deficiency in higher plants (Yang et al. 2014). We speculate that the expression of the iron complex transport system and the peptide/nickel transport system in epiphytic bacteria on female *S. thunbergii* plays an important role in mineral-deficient environments.

With respect to environmental adaptation, *trxb*, *TRR* and *rpoE* were enriched in the epiphytic bacteria on male *S. thunbergii*. The predicted genes *trxb* and *TRR* were reported to maintain intracellular redox balance (Xu et al. 2014), and *rpoE* can respond to changes in the external environment and regulate the transcription of genes related to environmental stress (Nuss et al. 2013). This adaptability is universal and not specific to a particular stress. However, *cspA* and *mcp* were more abundant in the epiphytic bacteria on female *S. thunbergii*. *cspA* belongs to the *cspA* family and is only related to low-temperature adaptation (Zhou et al. 2021; Zhang et al. 2021). Similarly, *mcp* is only related to chemotaxis, which plays an important role in the *in-situ* biodegradation of pollutants (Jiang et al. 2005). Whether this functional difference leads to the difference in environmental adaptability between epiphytic bacteria of male and female algae remains to be further studied.

For spore formation and development, *parA* and *soj* with high predicted functional abundance in the epiphytic bacteria of male *S. thunbergii* can inhibit sporulation and sporulation gene expression by inhibiting the accumulation of activator proteins to directly inhibit gene expression (Quisel and Grossman 2000; Donczew et al. 2016). On the contrary, *yfkt* and *cgeA*, which were more enriched in epiphytic bacteria on female *S. thunbergii*, were reported to be involved in spore germination and maturation (Paidhungat and Setlow 2000; Tu et al. 2020). These results suggest that there may be significant differences in the spore formation and development of the epiphytic bacteria between male and female *S. thunbergii*.

In conclusion, we believe that sex plays a role to some extent in the assemblage of the epiphytic bacterial communities of macroalgae. A large number of studies have shown that the assemblage of epiphytic bacterial communities in macroalgae is affected by both surrounding environment and the host themselves (Florez et al. 2017; Serebryakova et al. 2018). Pei et al. (2021) reported that environmental factors, such as nitrogen, phosphorus and different geographical location, can significantly affect the epiphytic bacterial communities on the macroalgae. Because the host macroalgae were colonized by "appropriate" bacteria from the bacterial pool in the surrounding environment, the bacterial community structure of the same species in different locations also has high variability (Roth-Schulze et al. 2018; Comba González et al. 2021). In addition, the physiology of the macroalgae can also contribute to the epiphytic microbial assemblage (Goecke et al. 2010; Weigel and Pfister 2019). The host-specificity of epiphytic microbial communities might be due to extracellular secondary metabolites secreted from host during their life cycle. Because the male and female algal bodies are the same species, the metabolic types and substances released are very similar, so the algae of two sexes share most epiphytic bacteria. However, the nutrients released by the male and female macroalgae around the cells are not exactly the same and the sex-specific algal nutrients attract the differences in the colonization and prediction functions of sex specific bacteria, so the sex of host algae has a certain contribution to the community structure of epiphytic bacteria.

From the overall findings in this study, it can be concluded that sex differentiation plays a minor role but not decisive factor in shaping the epiphytic bacterial communities on male and female *S. thunbergii*. The results from epiphytic bacterial functional analysis confirmed the above speculation. Our results enrich the knowledge system of the epiphytic bacteria community structure of male and female algae, which provides the basis for the study of the construction mechanism of macroalgae bacterial community of male and female *S. thunbergii*.

## Supplementary Information

**Additional file 1: Table S1.** Identification of the culturable epiphytic bacteria on both male and female *S. thunbergi.* **Table S2.** The proportions of epiphytic bacterial ASVs of male and female *S. thunbergii* at the phylum level. **Table S3.** The proportions of male- and female-specific epiphytic bacterial ASVs of male and female *S. thunbergii.* **Figure S1.** Sequencing depth of the epiphytic bacteria on male and female *S. thunbergii.* **Figure S2.** The structure of the epiphytic bacterial community associated with male and female *S. thunbergii* (A) All; (B) Abundant taxa; (C) rare taxa.

## Data Availability

The datasets generated and analyzed during the current study are available in the NCBI SRA repository under the BioProject ID: PRJNA656434 (Biosamples accession numbers SAMN15784712, Male: SRR16971116–SRR16971118, Female: SRR16971119–SRR16971121). Results for concurrent bacterial cultures are available in the NCBI SRA repository under the BioProject ID: PRJNA656434 (Male: SAMN23401982, Female: SAMN23401981).

## Abbreviations

*S. thunbergii*: *Sargassum thunbergii*
*P. haitanensis*: *Porphyra haitanensis*
*P. cathayana*: *Populus cathayana*
LDA: Linear discriminant analysis
