# Supplementary material for: Diversity of epiphytic bacterial communities on male and female Sargassum thunbergii
Source: AMB Express. 2022 Jul 16;12:97. doi: 10.1186/s13568-022-01439-1 (PMC9288574; doi:10.1186/s13568-022-01439-1)
Supplement: Supplementary file 1 — Additional file 1: Table S1. Identification of the culturable epiphytic bacteria on both male and female S. thunbergi. Table S2. The proportions of epiphytic bacterial ASVs of male and female S. thunbergii at the phylum level. Table S3. The proportions of male- and female-specific epiphytic bacterial ASVs of male and female S. thunbergii. Figure S1. Sequencing depth of the epiphytic bacteria on male and female S. thunbergii. Figure S2. The structure of the epiphytic bacterial community associated with male and female S. thunbergii (A) All; (B) Abundant taxa; (C) rare taxa. [file 13568_2022_1439_MOESM1_ESM.docx]

**Diversity of** **epiphytic bacterial communities on male and female *Sargassum thunbergii***

**Jing Wang^1^, Zhibo Yang^1^, Gaoge Wang^1^, Shuai Shang^1,2^, Xuexi Tang^1,3^, Hui Xiao^1,3*^.**

^1^ College of Marine Life Sciences, Ocean university of China, 266003 Qingdao, China

^2^ College of Biological and Environmental Engineering, Binzhou University, 256600 Binzhou, China

^3^ Laboratory for Marine Ecology and Environmental Science, Qingdao National Laboratory for Marine Science and Technology, 266000 Qingdao, China

*** Correspondence:**

Hui Xiao

xiaohui@ouc.edu.cn

**Supplementary Tables and Figures**

**Table S1. Identification of the culturable epiphytic bacteria on both male and female *S. thunbergii***

| **Phylum** | **Genus** | **Species** | **Male** | **Female** |
| --- | --- | --- | --- | --- |
| *Proteobacteria* | *Vibrio* | *Vibrio alginolyticus* | 5 | 11 |
|  |  | *Vibrio owensii* | 6 | 6 |
|  |  | *Vibrio neocaledonicus* | 5 | 3 |
|  |  | *Vibrio natriegens* | 1 | 1 |
|  |  | *Vibrio harveyi* | 3 | / |
|  |  | *Vibrio azureus* | 1 | / |
|  |  | *Vibrio parahaemolyticus* | 1 | / |
|  | *Providencia* | *Providencia rettgeri* | 1 | / |
|  | *Pseudoalteromonas* | *Pseudoalteromonas phenolica* | 1 | / |
|  |  | *Pseudoalteromonas flavipulchra* | 2 | 2 |
|  |  | *Pseudoalteromonas mariniglutinosa* | / | 1 |
|  |  | *Pseudoalteromonas shioyasakiensis* | 1 | / |
|  | *Halomonas* | *Halomonas aquamarina* | / | 1 |
|  | *Alteromonas* | *Alteromonas australica* | / | 2 |
|  |  | *Alteromonas macleodii* | / | 1 |
|  |  | *Alteromonas mediterranea* | / | 1 |
|  | *Idiomarina* | *Idiomarina sediminum* | / | 1 |
| *Firmicutes* | *Bacillus* | *Bacillus oceanisediminis* | 1 | / |
|  | *Exiguobacterium* | *Exiguobacterium aquaticum* | / | 1 |
| *Bacteroidetes* | *Tenacibaculum* | *Tenacibaculum mesophilum* | / | 2 |
| *Actinomycetes* | *Micrococcus* | *Micrococcus luteus* | 1 | / |
| **Proteobacteria* | **Pseudochrobactrum* | **Pseudochrobactrum saccharolyticum* |  | 1 |
| **Sum** | | | 29 | 34 |

Note: Asterisks (*) indicates novel mRNA species, based on 92.84% similarity of 16S rDNA sequence;

Backslash (/) indicates no bacteria were identified.

**Table S2. The proportions of epiphytic bacterial ASVs of male and female *S. thunbergii* at the phylum level**

| **Phylum** | **Male** | **Female** |
| --- | --- | --- |
| *Proteobacteria* | 0.747527399 | 0.773050601 |
| *Bacteroidetes* | 0.19884512 | 0.178383587 |
| *Actinobacteria* | 0.018222577 | 0.019538973 |
| *Planctomycetes* | 0.010178053 | 0.013786381 |
| *Patescibacteria* | 0.014251203 | 0.008445598 |
| *TM7* | 0.002411 | 0.002686759 |
| *Firmicutes* | 0.002825868 | 0.000781139 |
| *Acidobacteria* | 0.002057465 | 0.001236179 |
| *Thermi* | 0.001033286 | 0.000673439 |
| *Verrucomicrobia* | 0.000946777 | 0.000157427 |
| *GN02* | 0.000705195 | 0.000360334 |
| *Bacteria_unclassified* | 0.000270508 | 0.000309857 |
| *Spirochaetes* | 0.000149984 | 0.000200158 |
| *SBR1093* | 8.14201E-05 | 0.000157927 |
| *Cyanobacteria* | 0.000243189 | 0 |
| *Tenericutes* | 2.94612E-05 | 0.000127691 |
| *NKB19* | 7.36531E-05 | 2.99861E-05 |
| *Chlorobi* | 0 | 4.39797E-05 |
| *Chloroflexi* | 4.41919E-05 | 0 |
| *TM6* | 4.41919E-05 | 0 |
| *Lentisphaerae* | 0 | 2.99861E-05 |
| *Gemmatimonadetes* | 2.99969E-05 | 0 |
| *Nitrospirae* | 2.94612E-05 | 0 |

**Table S3. The proportions of male- and female-specific epiphytic bacterial ASVs of male and female *S. thunbergii***

| **Genus** | **Male** | **Female** |
| --- | --- | --- |
| *Reichenbachiella* | 0 | 0.0550413 |
| *Labrenzia* | 0 | 0.04200731 |
| *Spongiibacter* | 0 | 0.03828002 |
| *Thiothrix* | 0 | 0.03689277 |
| *Ahrensia* | 0 | 0.02376168 |
| *Chelativorans* | 0 | 0.02376168 |
| *Thalassospira* | 0 | 0.0217014 |
| *Nitrosomonas* | 0 | 0.01953166 |
| *Thermomonas* | 0 | 0.01953166 |
| *Cytophagales_unclassified* | 0 | 0.01736111 |
| *Roseivirga* | 0 | 0.01519138 |
| *Sphingopyxis* | 0 | 0.01519138 |
| *Pseudozobellia* | 0 | 0.01462118 |
| *Lutibacter* | 0 | 0.0138206 |
| *Ellin329_unclassified* | 0 | 0.00879575 |
| *Nisaea* | 0 | 0.00868056 |
| *Psychromonas* | 0 | 0.00742578 |
| *Oceaniserpentilla* | 0 | 0.00594063 |
| *Simiduia* | 0 | 0.00518283 |
| *OPB56_unclassified* | 0 | 0.00434028 |
| *Gaiellales_unclassified* | 0 | 0.00345494 |
| *Verrucomicrobiaceae_unclassified* | 0 | 0.00345494 |
| *NKB19_unclassified* | 0 | 0.00297031 |
| *Victivallaceae_unclassified* | 0 | 0.00297031 |
| *Polaribacter* | 0.23077867 | 0 |
| *Croceitalea* | 0.07167921 | 0 |
| *Coxiella* | 0.05837724 | 0 |
| *Psychrobacter* | 0.05242025 | 0 |
| *Amaricoccus* | 0.04908513 | 0 |
| *Desulfococcus* | 0.04039875 | 0 |
| *Cohaesibacteraceae_unclassified* | 0.04028136 | 0 |
| *Novosphingobium* | 0.03712276 | 0 |
| *Marinicella* | 0.03599104 | 0 |
| *Aquicella* | 0.03431004 | 0 |
| *Dinoroseobacter* | 0.02972491 | 0 |
| *Jannaschia* | 0.02515412 | 0 |
| *SM2F09_unclassified* | 0.02441487 | 0 |
| *Burkholderiales_unclassified* | 0.02042563 | 0 |
| *Devosia* | 0.01791577 | 0 |
| *Actibacter* | 0.01479659 | 0 |
| *028H05-P-BN-P5_unclassified* | 0.0134767 | 0 |
| *NB1-i_unclassified* | 0.0133172 | 0 |
| *Luteimonas* | 0.01271595 | 0 |
| *Comamonas* | 0.01138799 | 0 |
| *Puniceicoccaceae_unclassified* | 0.0112043 | 0 |
| *Caedibacter* | 0.01035753 | 0 |
| *Xanthomonadaceae_unclassified* | 0.00900627 | 0 |
| *Hahella* | 0.00887814 | 0 |
| *Pseudoxanthomonas* | 0.00755824 | 0 |
| *SHAB590_unclassified* | 0.00739785 | 0 |
| *Desulfarculaceae_unclassified* | 0.00604659 | 0 |
| *CCU21_unclassified* | 0.00546953 | 0 |
| *NB1-j_unclassified* | 0.00546953 | 0 |
| *Fusibacter* | 0.00443907 | 0 |
| *SJA-4_unclassified* | 0.00443907 | 0 |
| *TK17_unclassified* | 0.00443907 | 0 |
| *Pla3_unclassified* | 0.00364606 | 0 |
| *Gemm-2_unclassified* | 0.0030233 | 0 |
| *Desulfobacteraceae_unclassified* | 0.00295968 | 0 |
| *JTB248* | 0.00295968 | 0 |
| *Nitrospiraceae_unclassified* | 0.00295968 | 0 |
| *Porifericola* | 0.00295968 | 0 |
| *Pseudidiomarina* | 0.00295968 | 0 |
| Sum | 0.93994713 | 0.40991148 |

**
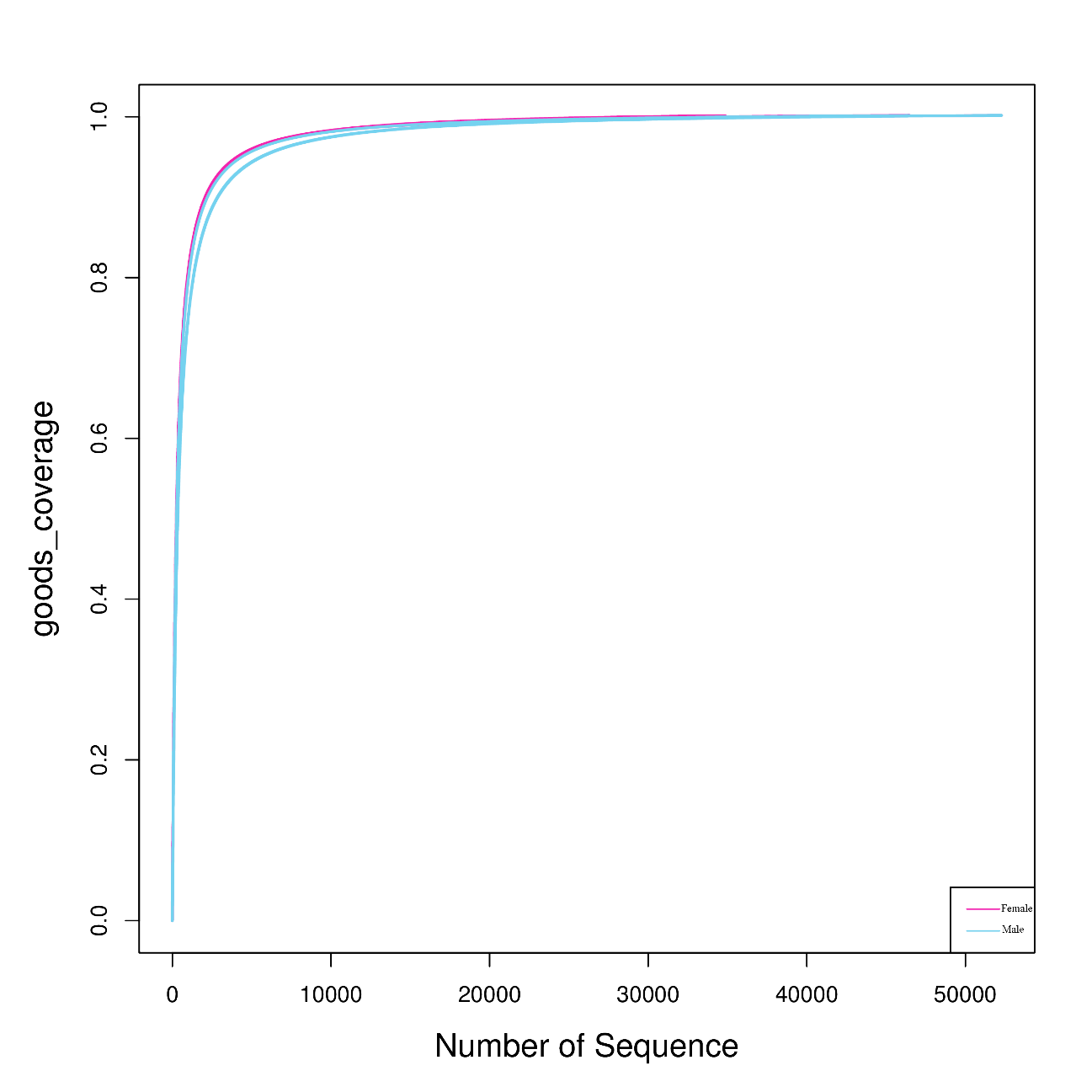
**

**Figure S1. Sequencing depth of the epiphytic bacteria on male and female *S. thunbergii.***

**
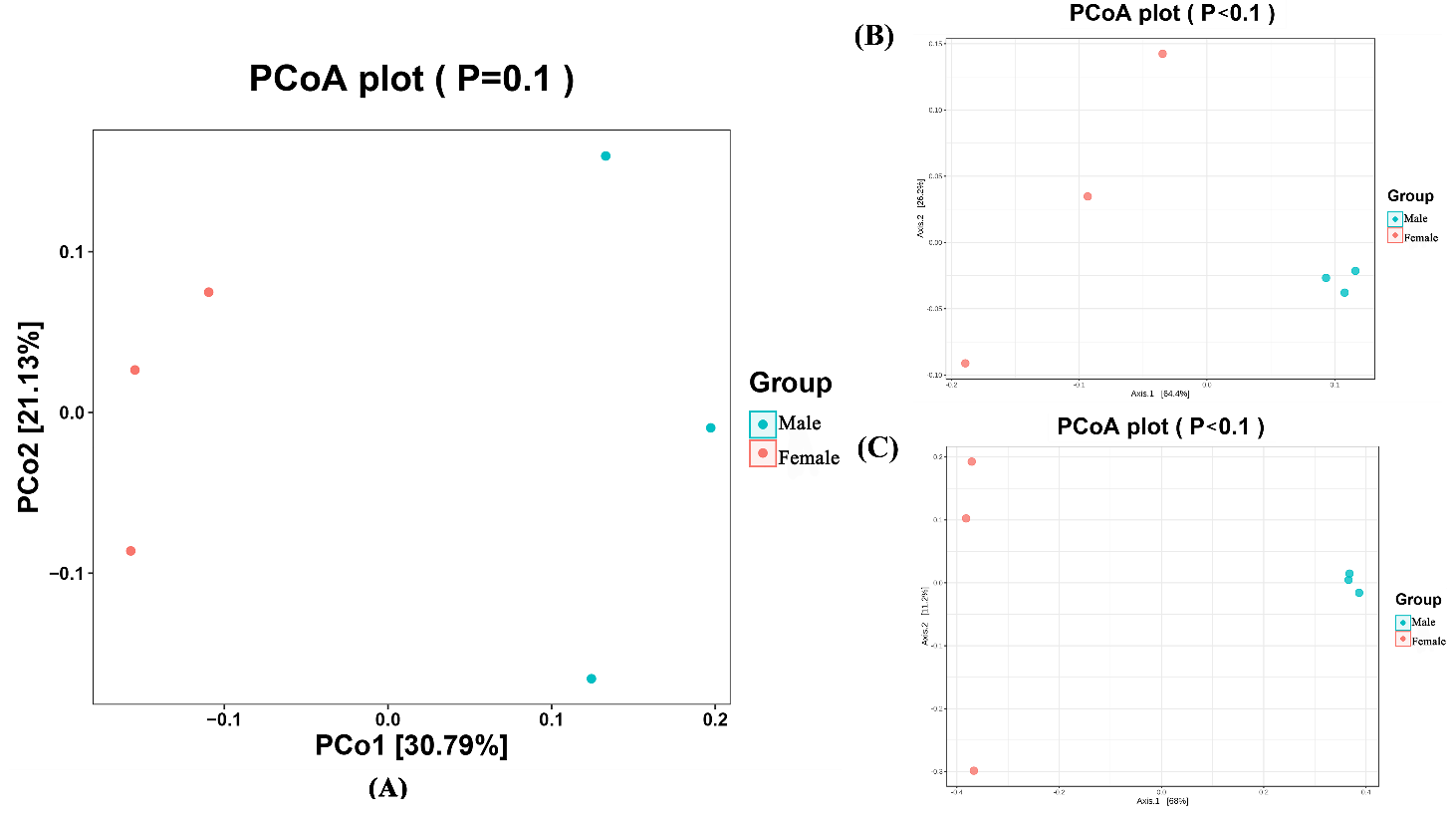
**

**Figure S2. The structure of the** **epiphytic bacterial community associated with male and female *S. thunbergii* (A)** **All; (B) Abundant taxa; (C)rare taxa**

Principal coordinate analysis (PCoA) plot of epiphytic bacterial communities of male and female *S. thunbergii* based on unweighted UniFrac distances. Colors represent male (blue) and female (red) *S. thunbergii*; determined by ANOSIM (A) R = 1, *P* = 0.12; (B) R = 1, *P* <0.1; (C) R = 1, *P* <0.1
